# The Rise and Fall of Osteopathy in Georgia

**Published:** 1900-01

**Authors:** 


### EDITORIAL NOTES AND COMMENTS.

The Business office of THE JOURNAL-RECORD is 305, 306 Fitten Building.

The Editorial office is 318-319-320 The Prudential.

Address all Business communications to Dr. M. B. Hutchins, Mgr.

Make remittances payable to THE ATLANTA JOURNAL-RECORD OF MEDICINE.

On matters pertaining to the Editorial and Original Communications address Dr. Bernard Wolff, Atlanta.

Reprints of original articles will be furnished at cost price. Requests for the same should always be made *on the manuscript.*

We will present, post-paid, on request, to each contributor of an original article, twenty (20) marked copies of THE JOURNAL-RECORD containing such article.

### THE RISE AND FALL OF OSTEOPATHY IN GEORGIA.

" The time has come, the walrus said,
To talk of many things."

SOMETHING less than a year ago a representative of the organized band of irregulars called osteopaths appeared in Atlanta. He was unobtrusive and it was some time before his presence was detected. His rise to notoriety was due not to a conquest of the people but to the fact that he was an emissary from the Holy Shrine at Kirksville, Missouri, a town whose *réclame* arose from its being the point of issuance of the pellucid stream of distilled science called osteopathy, and from being nearly wiped out by a tornado, a piece of retributive justice on the part of outraged nature.

He bore letters of high indorsement from distinguished citizens in an adjoining state—the type of the great man who gives laudatory testimonials to nostrums in order that the public may see his well-known lineaments and may cut out his autograph with a view to preserving it until such a time as it may be bartered for a cancelled postage stamp.

He found the land fat, succulent and fairly teeming in that variety of scaleless fish which ingests its nutriment without the aid of teeth, and he sent the glad tidings speeding home to Missouri. Then came others, equipped with the sinews of war to possess the land, by the same stratagems which had proven so successful in traducing the legislatures in the raw states of the West. A bill was introduced into the senate containing the same specious arguments and suspiciously moderate demands which won for osteopathy legal recognition elsewhere. The bill was referred to the general judiciary committee of the senate and was approved. Before being passed on by the senate a recommitment was secured and an opportunity given for the members of the regular profession to have a hearing. The members of the committee were convinced, or so expressed themselves, that the bill should not pass, and so voted *in committee.* It was then hoped that the bill had been killed "in a bornin'." But when it finally came up before the senate it passed with scarcely any opposition, even the members of the general judiciary committee, who had reported adversely, voting for it—some with shamefaced apologies.

A little later the bill came up in the House and passed so quickly that there was almost a suggestion that the unctuous properties of the coin of the republic had been utilized to lubricate its passage. It was even vaguely hinted that the blandishments of the fair but frail sisterhood were used to convince some of the more recalcitrant Solons of the great and shining merits of osteopathy, and their influence was thus secured for the bill.

The newspapers also fell under the ban of suspicion. Nothing adverse to osteopathy was allowed to appear. "The crowded condition of their news columns precluded any communication of a controversial nature." But the osteopaths had the floor. They seemed to be able to catch the Speaker's eye when he was blind to every one else. Then the osteopaths, seeing that the land had been delivered into their hand, lifted up their voices in loud and joyful pæans, and old women of both sexes rejoiced with them. But their joy was fleeting, and the triumphant laugh that showed their yellow fangs back to the coated circumvallations was set in a dry and frozen grin by the cold douche of the Governor's veto. Then they shoggled and scrambled back to their holes like so many fiddlercrabs, leaving "the sole representative" to tearfully explain why he was not a fraud and how it all happened, as if there was any one who wanted to know. Then there was silence, and nothing has been heard except the jingle of the bells on the cap of the evangelistic merry Andrew of Cartersville.

The osteopathic episode is now closed and State saved; but it behooves the profession to be on the alert, and not to sleep in fancied security while the chinch that has been overlooked in the joints of the bedstead has had time to recuperate and propagate.

